# Association between the index of nutritional quality and lipid profile in adult women

**DOI:** 10.1002/edm2.358

**Published:** 2022-07-20

**Authors:** Saheb Abbas Torki, Effat Bahadori, Soheila Shekari, Soroor Fathi, Maryam Gholamalizadeh, Naeemeh Hasanpour Ardekanizadeh, Bahareh Aminnezhad, Mina Ahmadzadeh, Mahtab Sotoudeh, Fatemeh Shafie, Samira Rastgoo, Farhad Vahid, Saeid Doaei

**Affiliations:** ^1^ Department of Nutrition, Faculty of Nutrition Sciences Shiraz University of Medical Sciences Shiraz Iran; ^2^ Mashhad University of Medical Sciences Mashhad Iran; ^3^ Department of Nutrition, Science and Research Branch Islamic Azad University Tehran Iran; ^4^ Nutrition Research Center, School of Nutrition and Food Sciences Shiraz University of Medical Sciences Shiraz Iran; ^5^ Department of Community Nutrition, School of Nutrition and Food Science Isfahan University of Medical Sciences Isfahan Iran; ^6^ Cancer Research Center Shahid Beheshti University of Medical Sciences Tehran Iran; ^7^ Department of Clinical Nutrition, School of Nutrition and Food Sciences Shiraz University of Medical Sciences Shiraz Iran; ^8^ Department of Clinical Nutrition and Dietetics, Faculty of Nutrition and Food Technology, National Nutrition and Food Technology Research Institute Shahid Beheshti University of Medical Sciences Tehran Iran; ^9^ Department of Clinical Nutrition National Nutrition and Food Technology Research Institute Faculty of Nutrition Sciences and Food Technology Shahid Beheshti University of Medical Sciences Tehran Iran; ^10^ Population Health Department Nutrition and Health Research Group Luxembourg Institute of Health Strassen Luxembourg; ^11^ Department of Community Nutrition National Nutrition and Food Technology Research Institute Faculty of Nutrition Sciences and Food Technology Shahid Beheshti University of Medical Sciences Tehran Iran

**Keywords:** biotin, dyslipidaemia, index of nutritional quality, lipid profile, niacin, nutritional assessment

## Abstract

**Background:**

Dyslipidaemia is a group of abnormalities that predispose people to heart disease. The index of nutritional quality (INQ) is a tool for qualitative and quantitative nutritional assessment, which has special significance in assessing clinical nutritional problems. The objective of this study was to determine the association between the INQ and lipid profile in adult women.

**Methods:**

This was a cross‐sectional study on 360 healthy women referring to the nutrition clinic of Shohadaye Tajrish hospital, Tehran, Iran. Calorie and nutrient intake were assessed using a validated food frequency questionnaire. The amount of physical activity was estimated using a validated International Physical Activity Questionnaire. To measure serum lipid levels, 5 ml of venous blood samples was taken from the participants.

**Results:**

The results showed a negative association between total cholesterol and the INQ of niacin (*B* = −0.110, *p* = .02) and between high‐density lipoprotein cholesterol with the INQ of biotin (*B* = −0.119, *p* = .01). Also, a positive association was found between triglyceride and the INQ of B6 (*B* = 0.096, *p* = .04). The results remained significant after adjusting for body mass index, waist circumference and total energy intake (except for niacin).

**Conclusions:**

Findings of the present study suggest that a diet rich in niacin and low in vitamin B6 and biotin may be associated with an improved lipid profile that reduces lipid‐related diseases such as fatty liver, metabolic syndrome and cardiovascular disease. Further studies are needed to confirm these findings and to identify the underlying mechanisms.

## Graphical abstract

Findings of the present study suggest that a diet rich in niacin and biotin and low in vitamin B6 may be associated with an improved lipid profile that reduces lipid‐related diseases such as fatty liver, metabolic syndrome, and cardiovascular disease.

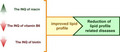

## 1 INTRODUCTION

Dyslipidaemia is a group of abnormalities that predispose people to heart disease by increasing the serum level of total cholesterol (TC) and low‐density lipoprotein cholesterol (LDL) and decreasing high‐density lipoprotein cholesterol (HDL).^1^ Dyslipidaemia is determined by TC, LDL, triglyceride (TG) and apolipoprotein B levels higher than the 90th percentile and/or HDL and apolipoprotein A1 levels below the 10th percentile of public people.^2^ The prevalence of dyslipidaemia is considerable in Iran, and hypertriglyceridemia was reported to be more prevalent in men whereas hypercholesterolemia, high LDL and low HDL were more prevalent in women.^3^ The American and European Cardiovascular Association recommends screening of all age groups to prevent the initial outbreak of cardiovascular disease.^4^, ^5^ All new guidelines emphasize on early detection of risk factors of CVDs such as imbalanced lipid profile and high blood pressure.^4^

Lifestyle risk factors including unhealthy diet, smoking, physical inactivity and obesity have a direct association with dyslipidaemia.^5^ For example, some diets, such as the Mediterranean diet or diets, that increase the gut‐friendly microbiota have positive effects on dyslipidemia.^6^, ^7^, ^8^ High levels of vitamin D in pregnancy were reported to improve lipid profile.^9^ Also, a study reported that higher blood levels of iron and copper are both associated with lower risks of lipid metabolism disorders and hyperlipidaemia. Furthermore, they are also associated with lower blood levels of total cholesterol and LDL cholesterol.^10^, ^11^ As an essential trace, copper is a part of antioxidant enzymes and have protective effect on dyslipidaemias. While some studies reported that serum copper was positively associated with TC and LDL cholesterol, thereby increasing the risk of dyslipidaemia.^12^, ^13^ In addition, there is evidence that high intake of some vitamins and minerals such as niacin, vitamin D, E, C and selenium play a protective role in the prevention of hyperlipidaemia.^14^, ^15^, ^16^, ^17^ However, recent study reported vitamin C supplementation had no significant effect on serum cholesterol, triglycerides and HDL.^19^

The role of dietary factors in chronic non‐communicable diseases is now well known.^20^ There are many ways to evaluate the quality of diets, and recent studies have considered dietary indices as better indicators than measuring the individual dietary components.^18^ The index of nutritional quality (INQ) is a tool for qualitative and quantitative nutritional assessment which has special significance in assessing clinical nutritional problems.^18^, ^21^, ^22^ This index is the ratio of nutrient intake to average intake according to the amount of dietary calories.^23^ The association between the INQ and some chronic diseases has been reported. For example, a recent study on the association between INQ and ulcerative colitis indicated that adequate intake of vitamin C and folic acid reduces the risk of ulcerative colitis.^24^ Another study on the relationship between the index of the INQ and non‐alcoholic fatty liver disease concluded that consuming a healthy diet, especially including high amounts of vitamin B1, B2, B3, B12, D and zinc reduced the risk of non‐alcoholic fatty liver disease.^22^ In addition, the results of the case–control study reported that INQ of some nutrients including vitamin C, iron, vitamin B6, pantothenic acid, selenium and magnesium was inversely associated with obesity.^25^ Due to the lack of study on the association between the INQ and hyperlipidaemia in adults, this study aimed to investigate the association between the INQ and lipid profile in Iranian adult women.

## 2 METHODS

This cross‐sectional study was performed on 360 Iranian adult women in Tehran, Iran. The sample size was obtained using Open EPI online software and the odds ratio obtained in similar previous studies. The participants were selected from healthy women referring to the nutrition clinic of Shohadaye Tajrish Hospital, Tehran, Iran.

The participants were included in the study if they were between 35 and 75 years old, have willing to participate in the study, not suffering from diseases affecting serum lipid levels and did not use cholesterol and lipid‐lowering drugs. Participants with alcohol or drug addiction (*n* = 2), smoking (*n* = 2), weight‐related illnesses (i.e., specific psychological or neurological disorders, insulin resistance, thyroid disease, liver disease, kidney failure, infectious diseases, history of multiple sclerosis (MS) and high blood pressure, people on dialysis) (*n* = 2) and pregnant or lactating women were excluded from the study (*n* = 1). A total of 353 women were included in the final analyses.

### 2.1 Anthropometric and physical activity assessments

In the next step, information about age, height, weight and body mass index (BMI) were collected through face‐to‐face interviews. The amount of physical activity was estimated using a validated International Physical Activity Questionnaire.

### 2.2 Lipid profile measurements

To measure serum lipid levels, 5 ml of venous blood samples was taken from the participants after 12 h of fasting, and serum levels of TG, TC, HDL and LDL were measured using the photometric method and quantitative diagnostic kits.

### 2.3 Nutritional assessments

The calorie intake and required nutritional information were obtained using a semi‐quantitative food frequency questionnaire (FFQ) that has already been validated in Iran. Individuals were asked to report the frequency of consumption of each food item according to its standard size over the last year. The data obtained from these questionnaires were analysed using Nutritionist‐IV software, and the average daily intake of energy and nutrients was calculated.

The INQ for each nutrient was calculated as the ratio of the amount of nutrient consumed per day per 1000 kcal to the recommended dietary allowance (RDA) of that nutrient per 1000 kcal. Adequate intake (AI) is defined for nutrients that were no specific RDA values. The participants were excluded from the study if <40% of the items in the FFQ were used last year, and finally, the analysis was performed on the remaining 360 individuals.

### 2.4 Statistical analysis

In order to compare different variables in individuals with normal and abnormal lipid profiles, independent t‐test methods (for quantitative variables) and Chi‐square (for qualitative variables) were used. To investigate the relationship between lipid profile and the INQ index, after classifying the level of lipid indices into normal and abnormal values, the logistic regression method was used with baseline measures as a covariate. IBM SPSS software (version 23) was used for statistical analysis of the data, and *p* value <.05 was considered statistically significant.

## 3 RESULTS

Table 1 shows the distribution of sociodemographic, anthropometric and lifestyle characteristics of the participants. The participants with hypercholesterolemia had higher age (51.74 ± 8.74 vs. 47.95 ± 7.71 y, *p* = .001) and higher BMI (29.97 ± 4.50 vs. 28.66 ± 4.02 kg/m^2^, *p* = .005) than the participants with normal serum cholesterol. Significant differences were observed between the two groups in height, TG, cholesterol, LDL, ALP (Alkaline phosphatase), SGOT (serum glutamic‐oxaloacetic transaminase) and SGPT (Serum glutamic pyruvic transaminase).

**TABLE 1:** Distribution of sociodemographic, anthropometric and lifestyle characteristics of the participants

	Normal serum cholesterol (*n* = 210)	Hypercholesterolemia (*n* = 143)	*p*
Age (years)	47.95 ± 7.71	51.74 ± 8.74	**.001**
Height (cm)	156.69 ± 5.65	155.40 ± 5.21	.028
Weight (kg)	70.476 ± 11.09	72.50 ± 12.10	.111
BMI (kg/m^2^)	28.66 ± 4.02	29.97 ± 4.50	.005
TG (mg/L)	109.26 ± 68.32	150.01 ± 73.65	.001
Cholesterol (mg/L)	168.47 ± 19.49	233.78 ± 24.69	.001
LDL (mg/L)	93.99 ± 19.91	144.60 ± 24.24	.001
SGOT (U/L)	18.27 ± 4.85	19.30 ± 5.42	.067
SGPT (U/L)	17.14 ± 7.20	19.55 ± 10.11	.015
ALP (U/L)	216.72 ± 71.20	237.21 ± 68.06	.007
GGT (U/L)	18.89 ± 13.19	21.63 ± 12.88	.053
Using alcohol			
Yes	3 (1.4%)	0 (0.0%)	.27
No	207 (98.6%)	143 (100.0%)	
Smoking			
Yes	3 (1.4%)	0 (0.0%)	.24
No	207 (98.6%)	142 (100.0%)	

Table 2 shows the distribution of daily intakes across normal cholesterol and hypercholesterolemia groups. There was no significant difference in the intake of dietary components between the two groups.

**TABLE 2:** Comparison of the dietary intake of the participants with normal serum cholesterol and hypercholesterolemia

	Normal serum cholesterol (*n* = 210)	Hypercholesterolemia (*n* = 143)	*p*
Energy (kcal/d)	2723 ± 729.48	2755 ± 1137	.88
Protein (g/d)	83.29 ± 24.53	90.86 ± 56.41	.450
CHO (g/d)	407.219 ± 117.962	395.724 ± 133.98	.682
Total fat (g/d)	90.99 ± 32.95	95.31 ± 52.18	.662
Cholesterol (mg/d)	235.56 ± 100.74	250.21 ± 161.10	.631
SAFA (g/d)	27.05 ± 9.68	32.15 ± 26.23	.267
MUFA (g/d)	30.19 ± 12.29	30.87 ± 14.72	.822
PUFA (g/d)	19.39 ± 8.39	19.43 ± 8.89	.983
Omega‐3 fatty acids (g/d)	1.19 ± 0.372	1.32 ± 0.727	.330
Omega‐6 fatty acids (g/d)	5.67 ± 7.35	5.17 ± 6.59	.745
Sodium (mg/d)	5654.53 ± 2204.49	5669.82 ± 2982.98	.979
Potassium (mg/d)	4003.22 ± 1320.23	4184.36 ± 2338.50	.675
Vitamin A (RAE/d)	512.86 ± 305.45	449.95 ± 201.07	.260
β‐carotene (μg/d)	3390.14 ± 2208.83	2769.13 ± 1571.19	.136
α‐carotene (μg/d)	643.73 ± 745.96	416.17 ± 330.21	.066
Lutein (μg/d)	1614.20 ± 856.50	1472.95 ± 712.70	.412
β‐Cryptoxanthin (μg/d)	309.70 ± 175.68	281.66 ± 179.57	.475
Lycopene (μg/d)	7966.31 ± 4477.00	7180.53 ± 4739.02	.442
Vitamin C, mg/day	141.02 ± 77.32	163.04 ± 148.35	.416
Calcium (mg/d)	1162.65 ± 402.59	1423.88 ± 1453.73	.295
Iron (mg/d)	20.209 ± 6.24	19.23 ± 6.80	.502
Vitamin D (μg/d)	1.08 ± 0.819	1.014 ± 0.880	.724
Vitamin E (mg/d)	18.19 ± 11.84	17.02 ± 11.05	.642
Thiamin (mg/d)	2.31 ± 0.824	2.42 ± 1.12	.641
Riboflavin (mg/d)	2.17 ± 0.697	2.47 ± 1.91	.366
Niacin (mg/d)	25.08 ± 8.09	23.34 ± 7.70	.319
Vitamin B6 (mg/d)	1.78 ± 0.564	1.86 ± 1.00	.677
Folate (total) (μg/d)	674.21 ± 194.92	672.68 ± 220.28	.974
Folate (DFE) (μg/d)	831.70 ± 276.55	811.76 ± 296.64	.754
Vitamin B12 (μg/d)	3.58 ± 2.08	4.37 ± 5.17	.388
Biotin (mg/d)	31.53 ± 11.63	30.77 ± 18.24	.826
Pantothenic acid (mg/d)	5.12 ± 1.77	5.54 ± 3.54	.517
Vitamin K (μg/d)	122.83 ± 56.90	126.33 ± 53.22	.773
Phosphorus (mg/d)	1371.96 ± 474.57	1551.15 ± 1330.41	.439
Magnesium (mg/d)	378.20 ± 138.32	387.30 ± 185.15	.804
Zinc (mg/d)	10.74 ± 4.00	12.15 ± 7.90	.329
Copper (μg/d)	1.94 ± 0.592	1.87 ± 0.629	.586
Manganese (mg/d)	6.04 ± 3.15	5.42 ± 1.74	.261
Selenium (μg/d)	100.30 ± 44.26	96.82 ± 36.49	.694
Fluorine (μg/d)	3615.68 ± 2092.13	3069.10 ± 1550.06	.173
Chromium (μg/d)	0.028 ± 0.113	0.015 ± 0.043	.454
Fibre (total) (g/d)	30.32 ± 11.75	27.74 ± 10.72	.296
Fibre (soluble) (g/d)	1.17 ± 0.983	0.906 ± 0.537	.119
Fibre (insoluble) (g/d)	5.90 ± 4.27	4.80 ± 2.97	.170
Fibre (crude) (g/d)	12.37 ± 6.90	10.15 ± 6.17	.123
Sugar (total) (g/d)	147.91 ± 65.02	136.76 ± 63.31	.431
Glucose (g/d)	22.79 ± 8.80	19.85 ± 8.94	.137
Galactose (g/d)	3.19 ± 2.48	5.69 ± 13.02	.256
Fructose (g/d)	28.77 ± 11.59	25.00 ± 10.34	.120
Sucrose (g/d)	51.46 ± 29.69	46.16 ± 23.53	.365
Lactose (g/d)	11.90 ± 7.69	18.60 ± 35.11	.261
Maltose (g/d)	3.07 ± 1.60	2.88 ± 1.49	.591
Caffeine (mg/d)	203.51 ± 123.54	167.41 ± 91.51	.128

Table 3 shows the association between INQ and lipid profile. After controlling for several covariates, inverse associations were observed between TC and INQ of niacin (*B* = −0.110, *p* = .02), as well as HDL and INQ of biotin (*B* = −0.119, *p* = .01; *B* = −.167, *p* ≤ .01). A positive association was also identified between TG and INQ of vitamin B6 (*B* = 0.096, *p* = .04; *B* = 0.156, *p* = .01).

**TABLE 3:** Association between lipid profile and the index of nutritional quality (INQ) of the nutrients^a^

	TC		TG		HDL		LDL	
	*B*	*p*‐value	*B*	*p*‐value	*B*	*p*‐value	*B*	*p*‐value
Vitamin A	−0.038 −0.039	.42 .53	−0.033 0.003	.48 .96	−0.034 −0.088	.47 .16	0.015 0.011	.74 .85
Iron	−0.017 0.000	.71 .99	0.020 −0.015	.67 .80	−0.009 0.026	.84 .68	−0.029 −0.016	.53 .80
Calcium	0.020 0.028	.66 .65	0.011 0.043	.81 .50	0.052 −0.001	.27 .99	0.028 0.018	.55 .78
Magnesium	−0.077 −0.081	.10 .20	−0.016 −0.053	.73 .40	−0.035 −0.035	.45 .57	−0.040 −0.023	.40 .71
Phosphorus	−0.037 −0.022	.44 .72	0.009 0.015	.85 .81	0.005 −0.039	.92 .53	0.022 0.017	.64 .78
Zinc	−0.053 −0.046	.26 .46	0.075 0.112	.11 .07	−0.069 −0.103	.14 .10	−0.021 −0.036	.65 .56
Copper	−0.018 0.014	.70 .82	−0.007 −0.027	.88 .67	−0.084 −0.028	.07 .65	0.011 0.053	.82 .40
Manganese	−0.022 0.004	.64 .95	0.021 0.029	.66 .65	−0.042 −0.051	.37 .41	−0.007 0.018	.88 .77
Selenium	0.014 0.004	.76 .95	−0.010 −0.049	.83 .43	0.004 0.033	.90 .60	−0.019 0.013	.70 .63
Vitamin E	−0.026 −0.050	.58 .42	0.029 0.005	.54 .94	0.058 0.082	.21 .19	−0.073 −0.078	.12 .21
Thiamin	0.030 0.036	.52 .57	−0.054 −0.090	.25 .15	0.012 0.023	.80 .71	0.030 0.068	.53 .27
Riboflavin	0.010 0.000	.84 .99	0.014 −0.009	.75 .89	0.042 0.024	.37 .70	0.007 0.002	.89 .92
Niacin	−0.110 −0.085	.02 .18	0.026 0.027	.58 .67	−0.065 −0.045	.17 .47	−0.072 −0.077	.12 .21
Vitamin B6	0.007 0.057	.88 .36	0.096 0.156	.04 .01	−0.057 −0.051	.23 .41	−0.002 0.039	.95 .53
Folate	−0.008 0.022	.86 .72	0.069 0.019	.14 .76	−0.012 0.040	.80 .52	−0.009 0.061	.85 .33
Vitamin B12	−0.030 −0.006	.53 .92	0.029 0.049	.54 .44	−0.005 −0.036	.91 .56	0.001 0.004	.98 .95
Pantothenic acid	−0.011 0.024	.82 .70	0.021 0.069	.65 .27	−0.033 −0.037	.48 .55	0.024 0.016	.62 .80
Biotin	−0.053 −0.073	.25 .24	0.052 0.042	.27 .50	−0.119 −0.167	.01 <.01	−0.035 −0.040	.46 .52
Vitamin C	0.045 0.062	.34 .32	0.008 0.014	.86 .83	−0.010 −0.005	.83 .94	0.033 0.067	.48 .28
Vitamin D	−0.021 −0.009	.65 .88	−0.034 −0.040	.47 .52	0.018 −0.010	.70 .87	−0.006 −0.005	.89 .94
Vitamin K	−0.062 −0.061	.19 .33	0.019 0.028	.70 .66	−0.018 −0.029	.70 .64	0.019 −0.002	.70 .97

Abbreviations: HDL, high density lipoprotein; LDL, low density lipoprotein; TC, total cholesterol; TG, triglyceride.

^a^ First model controlled for age and second model controlled for age, BMI, WC and total energy intake.

## 4 DISCUSSION

To the best of our knowledge, this study is the first one to investigate the relationship between INQ and lipid profile in adult women. Our results showed negative associations between TC and INQ of niacin and HDL with INQ of biotin. A positive association was also found between TG and INQ of B6. There was no significant difference in the intake of dietary components between the two groups.

A number of studies, in line with our findings, have reported the role of niacin in improving lipid profile,^26^ dyslipidaemia and heart disease.^27^ Niacin supplementation improves the level of serum cholesterol and has a preventive effect on hepatic stasis.^28^ Ganji et al. reported that niacin reduces lipid accumulation in the liver. Possible mechanisms of the effect of niacin on hypercholesterolemia are inhibition of triglyceride transferase 2 and NADPH oxidation, which play key roles in fat metabolism.^29^ In fact, niacin may inhibit the release of free fatty acid (FAA) from adipose tissue which in turn reduces the activity of peroxisome proliferator‐activated receptor γ coactivator, Apo‐CIII (an inhibitor of lipoprotein lipase) and the accumulation of TG‐rich VLDL (very low‐density lipoprotein) in the liver. Consequently, reductions in VLDL lead to lower LDL levels and increase intracellular degradation of ApoB.^30^

Inconsistent with the positive association identified between TG and the INQ of vitamin B6 in this study. A study by Sani Hlais et al. reported that vitamin B6 supplementation reduced lipid profiles, including total cholesterol, by 10%.^31^ Furthermore, some studies have reported that vitamin B6 plays an important role in reducing fat accumulation in the liver.^32^ Kobayashi et al. reported that therapy with vitamin B6 as an inexpensive agent with few side effects decreased hepatic lipid accumulation, despite no significant changes in BMI.^33^ Recent studies with healthy men and women indicated that marginal vitamin B6 deficiency, led to significant reductions of arachidonic acid, eicosapentaenoic acid and docosahexaenoic acid concentrations in plasma and increase the plasma (*n*−6): (*n*−3) polyunsaturated fatty acids ratio, which in turn, can change the lipid profile.^36^ Animal studies have also shown an increase in total cholesterol, LDL and TG in rats with subnormal vitamin B6 status.^37^, ^38^ However, in the study of Ferro et al.,^34^ a positive association was found between liver fat and vitamin B6, which may reflect a higher consumption of high red meat, which is high in vitamin B6.^35^ Various factors such as faulty eating habits, low dietary diversity, work pressure and lifestyle all contribute to hyperlipidaemia and high blood fat levels.^39^ Contradictory results may be due to not considering the effect of some of these factors on the results in various studies.

The exact mechanism of action of vitamin B6 on fat is unclear. B6 plays a role in the elongation of fatty acids, methylation of phospholipids and movement of unsaturated fatty acids from triglycerides to phospholipids.^42^Vitamin B6 may decrease lipid profile by increasing the synthesis of prostaglandin E1.^43^

There is evidence that pharmacological doses of biotin decrease plasma lipid concentrations and modify lipid metabolism.^42^ Our results identified inverse associations between HDL and INQs of biotin. Inconsistent with our study, a randomized clinical trial reported that biotin treatment significantly reduced plasma TG and VLDL concentrations in the nondiabetic participants but had no significant effects on cholesterol in either the diabetic or nondiabetic subjects.^43^ In addition, a study reported that in biotin‐deficient rats the serum level of totally free and esterified cholesterol were lower than control or supplemented rats. In this study, HDL fractions of deficient rats did not change, but serum LDL fractions increased and VLDL fractions decreased.^44^ Studies on humans and rodents demonstrate that the administration of biotin at pharmacological doses decreases serum concentrations of triglycerides,^43^, ^45^ cholesterol^46^, ^47^ and FFA.^48^, ^49^ The mechanisms of these effects are still being elucidated. Previous studies have suggested that the hypotriglyceridemia effects of pharmacological concentrations of biotin are associated with decreased mRNA expression of lipogenic genes, such as the beta‐reduction pathway enzymes (i.e., acetyl‐CoA carboxylase and fatty acid synthase).^48^, ^49^, ^50^, ^51^ Other studies revealed that 8 weeks of dietary biotin supplementation increased the expression of the active T172‐AMPK protein levels in the liver and adipose tissue. Activation of hepatic AMPK decreases de novo lipogenesis.^48^, ^50^

This study has several strengths; the present study is the first one to report the association between INQ and lipid profile in adult women. In addition, the INQ estimates the intake of nutrients and energy according to the standard values, which may be a better method than the older methods which was used to estimate the intake of nutrients.^24^ However, the present study had some limitations; similar to other case–control studies, there is a possibility of recall bias. In addition, the INQ is based on DRI, which does not include some dietary substances.

## 5 CONCLUSION

The results of our research suggest that a diet rich in niacin and low in vitamin B6 and biotin may be associated with improved lipid profiles that reduce lipid‐related diseases such as fatty liver, metabolic syndrome and cardiovascular disease. Further studies are needed to confirm these findings and identify the underlying mechanisms.

## AUTHOR CONTRIBUTIONS

**Saheb Abbas Torki:** Software (equal). **Effat Bahadori:** Software (equal). **Soheila Shekari:** Validation (equal). **soroor Fathi:** Validation (equal). **Maryam Gholamalizadeh:** Writing – original draft (equal). **Bahareh Aminnezhad:** Software (equal). **Samira Rastgoo:** Investigation (equal).

## FUNDING INFORMATION

Funding for this study was provided by Cancer Research Center, Shahid Beheshti University of Medical Sciences, Tehran, Iran.

## CONFLICT OF INTEREST

The authors declare that they have no competing interests.

## ETHICS STATEMENT

All patients signed an informed consent form at baseline. This study was approved by the ethical committee of cancer research center, Shahid Beheshti University of Medical Sciences, Tehran, Iran (code: IR.SBMU.PHNS.REC.1399.038).

## Data Availability

Not applicable.
